# Prognostic significance of changes in heart rate following uptitration of beta-blockers in patients with sub-optimally treated heart failure with reduced ejection fraction in sinus rhythm versus atrial fibrillation

**DOI:** 10.1007/s00392-018-1409-x

**Published:** 2019-01-04

**Authors:** Ify R. Mordi, Bernadet T. Santema, Mariëlle Kloosterman, Anna-Maria Choy, Michiel Rienstra, Isabelle van Gelder, Stefan D. Anker, John G. Cleland, Kenneth Dickstein, Gerasimos Filippatos, Pim van der Harst, Hans L. Hillege, Marco Metra, Leong L. Ng, Wouter Ouwerkerk, Piotr Ponikowski, Nilesh J. Samani, Dirk J. van Veldhuisen, Aeilko H. Zwinderman, Faiez Zannad, Adriaan A. Voors, Chim C. Lang

**Affiliations:** 1 Division of Molecular and Clinical Medicine, Medical Research Institute, Mailbox 2, Ninewells Hospital & Medical School, University of Dundee, Dundee, DD1 9SY UK; 2 Department of Cardiology, University of Groningen, University Medical Center Groningen, Groningen, The Netherlands; 3 Division of Cardiology (CVK), and Berlin-Brandenburg Center for Regenerative Therapies (BCRT), German Centre for Cardiovascular Research (DZHK) partner site Berlin, Charité Universitätsmedizin Berlin, Berlin, Germany; 4 National Heart and Lung Institute, Royal Brompton & Harefield Hospitals, Imperial College, London, UK; 5 University of Bergen, Bergen, Norway; 6 Stavanger University Hospital, Stavanger, Norway; 7 National and Kapodistrian University of Athens, School of Medicine, Athens, Greece; 8 Department of Cardiology, Heart Failure Unit, Athens University Hospital Attikon, Athens, Greece; 9 Department of Medical and Surgical Specialties, Radiological Sciences and Public Health, Institute of Cardiology, University of Brescia, Brescia, Italy; 10 Department of Cardiovascular Sciences, University of Leicester, Glenfield Hospital, Leicester, UK; 11 NIHR Leicester Biomedical Research Centre, Glenfield Hospital, Leicester, LE3 9QP UK; 12 Department of Clinical Epidemiology, Biostatistics, and Bioinformatics, Academic Medical Centre, University of Amsterdam, Amsterdam, The Netherlands; 13 Department of Heart Diseases, Wroclaw Medical University, Wrocław, Poland; 14 Cardiology Department, Military Hospital, Wrocław, Poland; 15 Department of Epidemiology, Biostatistics and Bioinformatics, Academic Medical Center, Inserm CIC 1433, Amsterdam, The Netherlands; 16 Inserm CIC-P 1433, Université de Lorraine, CHRU de Nancy, FCRIN INI-CRCT, Nancy, France; 17 Department of Cardiology, Universitätsmedizin Göttingen (UMG), Göttingen, Germany; 18 National Heart Centre Singapore, 5 Hospital Drive, Singapore, Singapore

**Keywords:** Heart failure, Heart rate, Atrial fibrillation, Beta-blockers

## Abstract

**Background:**

In patients with heart failure with reduced ejection fraction (HFrEF) on sub-optimal doses of beta-blockers, it is conceivable that changes in heart rate following treatment intensification might be important regardless of underlying heart rhythm. We aimed to compare the prognostic significance of both achieved heart rate and change in heart rate following beta-blocker uptitration in patients with HFrEF either in sinus rhythm (SR) or atrial fibrillation (AF).

**Methods:**

We performed a post hoc analysis of the BIOSTAT-CHF study. We evaluated 1548 patients with HFrEF (mean age 67 years, 35% AF). Median follow-up was 21 months. Patients were evaluated at baseline and at 9 months. The combined primary outcome was all-cause mortality and heart failure hospitalisation stratified by heart rhythm and heart rate at baseline.

**Results:**

Despite similar changes in heart rate and beta-blocker dose, a decrease in heart rate at 9 months was associated with reduced incidence of the primary outcome in both SR and AF patients [HR per 10 bpm decrease—SR: 0.83 (0.75–0.91), *p* < 0.001; AF: 0.89 (0.81–0.98), *p* = 0.018], whereas the relationship was less strong for achieved heart rate in AF [HR per 10 bpm higher—SR: 1.26 (1.10–1.46), *p* = 0.001; AF: 1.08 (0.94–1.23), *p* = 0.18]. Achieved heart rate at 9 months was only prognostically significant in AF patients with high baseline heart rates (*p* for interaction 0.017 vs. low).

**Conclusions:**

Following beta-blocker uptitration, both achieved and change in heart rate were prognostically significant regardless of starting heart rate in SR, however, they were only significant in AF patients with high baseline heart rate.

## Introduction

Heart rate is a risk factor in patients with heart failure with reduced ejection fraction (HFrEF) that, when reduced, provides outcome benefits [1, 2]. However, the benefit of heart rate-mediated reduction is less clear in atrial fibrillation (AF). Studies in patients with HFrEF and AF have provided conflicting results, with some suggesting that elevated heart rate is associated with adverse outcome in HFrEF patients in AF, while others found no significant relationship [3–5]. Conceptually, reducing heart rate should have prognostic benefit in HFrEF patients in AF. Randomised controlled trials evaluating rate control strategies in patients with AF have only included small numbers of patients with HFrEF [6]. Additionally, very few studies have evaluated the importance of changes in heart rate over time [7, 8]. Despite the lack of data, current guidelines recommend an optimal heart rate between 60 and 100 bpm in patients with AF and HFrEF, while studies evaluating rate control in patients with AF (but not necessarily HFrEF) suggest that rates up to 110 bpm may be acceptable [6, 9].

One strategy for reducing heart rate is the use of beta-blockers, a mainstay of therapy in HFrEF [9, 10]. Although beta-blockers are prognostically beneficial in patients with HFrEF, it is unclear whether the beta-blocker-mediated reduction in heart rate directly affects prognosis, with several studies reporting conflicting results [11–17]. Furthermore, questions have recently been raised about the prognostic benefits of beta-blocker therapy in HFrEF patients with AF [18, 19]. In particular, there is very little information about whether increasing beta-blocker therapy in patients on sub-optimal doses might derive greater benefit from any associated heart rate reduction [20]. Despite the current uncertainty over the benefits of beta-blockers in HFrEF patients in AF, current guidelines recommend uptitration of beta-blocker therapy to the same target doses irrespective of the underlying heart rhythm.

To the best of our knowledge, the relative effects of change in heart rate following intensification of beta-blocker therapy have not been previously examined. Given the frequent co-existence of AF and HFrEF, it is important to determine whether patients in AF derive the same benefit from heart rate reduction and beta-blocker uptitration as those in SR, and whether this effect is modulated by changes in beta-blocker dose. We utilised the systems BIOlogy Study to Tailored Treatment in Chronic Heart Failure (BIOSTAT-CHF) dataset to compare the prognostic importance of changes in heart rate following beta-blocker uptitration in HFrEF patients in AF versus those in sinus rhythm (SR).

## Methods

### Patient selection

The BIOSTAT-CHF study design has been published previously [21]. Briefly, BIOSTAT-CHF was a large European, multi-center, multi-national, prospective, observational study of 2516 patients with new onset or worsening HF with either a left ventricular ejection fraction (LVEF) of ≤ 40% or plasma concentrations of Brain Natriuretic Peptide (BNP) > 400 pg/ml and/or N-terminal pro Brain Natriuretic Peptide (NT-proBNP) > 2000 pg/ml, who were being treated with furosemide ≥ 40 mg/day (or equivalent) and were on ≤ 50% of the target dose of angiotensin-converting enzyme inhibitor (ACEI)/angiotensin II receptor blocker (ARB) or beta-blocker therapy. Patients were recruited from both the in-patient and out-patient settings. Patients were classified as having AF if they had AF on their electrocardiogram (ECG) at their baseline visit and were reclassified at the second visit ECG. We excluded patients with paced or undetermined ECG rhythms and those with LVEF ≥ 40%.

In the first 3 months after recruitment, treating clinicians aimed to initiate and/or uptitrate ACEI/ARBs and beta-blockers to recommended target doses which have been previously published by the European Society of Cardiology [22]. Reasons for failure to successfully uptitrate and side effects have been previously published [23]. Following the 3-month uptitration period, patients entered a 6-month maintenance period where no further uptitration was mandated unless clinically indicated. Patients then were invited for a second visit at 9 months. The trial was approved by the local ethics committee of the participating centers and all patients provided written informed consent. The study complied with the Declaration of Helsinki.

### Clinical outcomes

Heart rate and rhythm were assessed by ECG with all patients supine and rested for at least 5 min. In BIOSTAT-CHF, all patients were followed up for clinical outcomes. After the scheduled visits at baseline and 9 months, patients were contacted by telephone every 6 months.

The primary outcome for this study was the combined endpoint of all-cause mortality or HF hospitalisation. HF hospitalisation was determined as admission to hospital ≥ 24 h due to worsening HF requiring either intravenous or increased dose of oral diuretics.

### Statistical analysis

Clinical, ECG and echocardiographic data was obtained at baseline, with clinical and ECG data obtained at 9 months. Normally distributed continuous variables were reported as mean ± SD and categorical data, as number with percentage in brackets. Comparisons between continuous variables were carried out using a two-tailed Student *t* test and categorical variables were tested using the Chi square test. Heart rate and beta-blocker dose at baseline and 9 months were analysed for their association with the primary outcome and all-cause mortality using the Cox proportional hazard model and Kaplan–Meier analysis. Competing risks regression with death as a competing risk was used to determine hazard ratios for hospitalisation alone. To adjust for treatment indication bias inverse probability weighting was used, the method of which has been explained in detail previously [23]. Variables included in the inverse probability weighting were age, baseline heart rate and country of origin.

Variables were tested for univariable significance and were then included in a multivariable model with the BIOSTAT-CHF risk score [23] to assess their independent association with outcome. SR and AF patients were evaluated separately and interaction testing between SR and AF was also performed within the whole cohort. Heart rate in increments of 10 bpm and beta-blocker dose as a percentage of target dose were examined. Increments of 12.5% of target beta-blocker dose were chosen to reflect clinically used dosages—for example, bisoprolol has a target dose of 10 mg, and is commonly increased in doses of 1.25 mg (12.5% of target dose). Nine-month outcomes only included patients who did not have an event in the first 9 months and those who had ECG data available. Correlations were assessed using Pearson correlation. A *p* value < 0.05 was considered significant throughout. Statistical analysis was performed using R version 3.4.1.

## Results

### Baseline characteristics

**Fig. 1:**
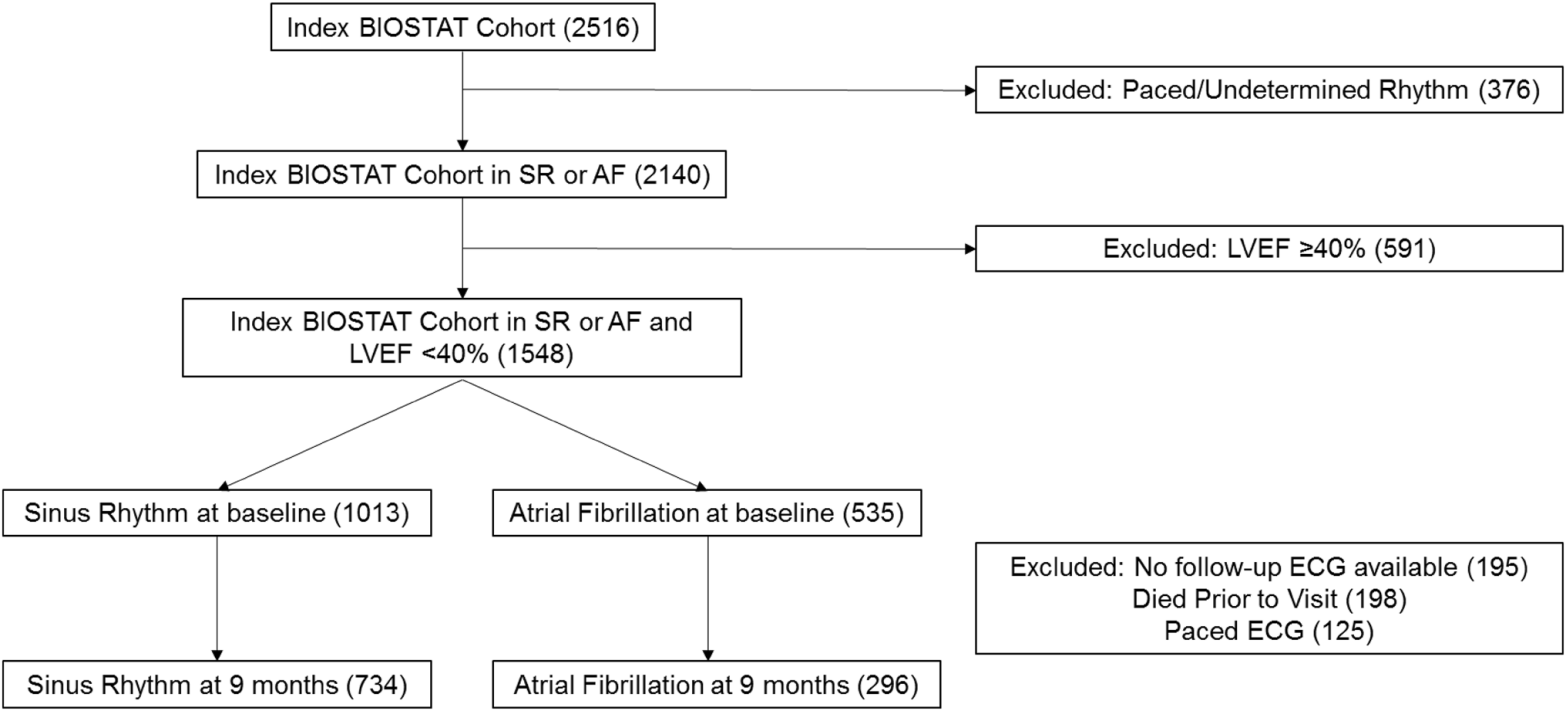
Cohort derivation. Derivation of the cohort from the BIOSTAT-CHF study

The baseline characteristics of the BIOSTAT-CHF study have been reported previously [21]. Median follow-up in BIOSTAT-CHF was 21 months. Derivation of the cohort for this study is shown in Fig. 1. In total, following exclusion of patients with LVEF ≥ 40% and paced or undetermined ECG rhythms, we included 1548 patients from the BIOSTAT-CHF index cohort (Table 1). 535 patients (34.6%) were in AF on their baseline ECG.

**Table 1:** Baseline cohort characteristics according to heart rhythm at baseline

	Total cohort (*n* = 1548)	Sinus rhythm (*n* = 1013)	Atrial fibrillation (*n* = 535)	*p* value between SR and AF
Age (years)	67 ± 12	65 ± 13	71 ± 10	< **0.001**
Men	1175 (75.9)	750 (74.0)	425 (79.4)	**0.018**
SBP (mmHg)	124 ± 21	124 ± 21	124 ± 21	0.55
DBP (mmHg)	76 ± 12	75 ± 12	76 ± 12	0.14
Heart rate (bpm)	83 ± 21	78 ± 17	93 ± 24	< **0.001**
QRS duration (ms)	112 ± 29	113 ± 29	112 ± 28	0.56
NYHA class^a^				< **0.001**
I	37 (2.4)	30 (3.0)	7 (1.3)	
II	557 (36.7)	400 (40.5)	157 (29.7)	
III	734 (48.4)	448 (45.3)	286 (54.2)	
IV	188 (12.4)	110 (11.1)	78 (14.8)	
Ischaemic aetiology	718 (47.4)	510 (51.4)	208 (39.8)	< **0.001**
Hypertension	935 (60.4)	609 (60.1)	326 (60.9)	0.76
Current smoker	252 (16.3)	201 (19.9)	51 (9.6)	< **0.001**
Diabetes mellitus	490 (31.7)	322 (31.8)	168 (31.4)	0.88
COPD	259 (16.7)	163 (16.1)	96 (17.9)	0.35
Renal impairment	357 (23.1)	193 (19.1)	165 (30.8)	< **0.001**
ACEI/ARB	1158 (74.8)	770 (76.0)	388 (72.5)	0.13
Beta-blocker	1299 (83.9)	853 (84.2)	446 (83.4)	0.67
Beta-blocker dose %				< **0.001**
0	250 (16.1)	161 (15.9)	89 (16.6)	
1–49	938 (60.6)	644 (63.6)	294 (55.0)	
50–99	292 (18.9)	176 (17.4)	116 (21.7)	
≥ 100	68 (4.4)	32 (3.2)	36 (6.7)	
MRA	860 (55.6)	575 (56.8)	285 (53.3)	0.19
Digoxin	284 (18.3)	86 (8.5)	198 (37.0)	< **0.001**
LVEF (%)	27.3 ± 6.9	27.1 ± 7.0	27.8 ± 6.9	0.07

Bold values indicate *p* < 0.05

32 patients (2.1%) did not have NYHA class recorded

*SBP* systolic blood pressure, *DBP* diastolic blood pressure, *COPD* chronic obstructive pulmonary disease, *ACEI* angiotensin-converting enzyme inhibitor, *ARB* angiotensin receptor blocker, *LVEF* left ventricular ejection fraction, *NT-proBNP* N-terminal pro B-type natriuretic peptide

^a^Median (interequartile range)

### Relationship between baseline heart rate and outcome

In total, the primary outcome occurred in 554 patients [35.8% of the total cohort; 323 (31.8%) in SR and 231 (43.2%) in AF], including 324 deaths [20.9% of the total cohort; 212 (18.6%) in SR and 112 (28.0%) in AF] and 337 hospitalisations [21.8% of the total cohort; 198 (19.5%) in SR and 139 (26.0%) in AF] (Table 2).

**Table 2:** Cox regression analyses of baseline heart rate on the primary outcome of mortality and heart failure hospitalisation

	Sinus rhythm (*n* = 1013)			Atrial fibrillation (*n* = 535)				Interaction *p* value
	Number of events (%)	Multivariable hazard ratio (95% CI)	*p* value	Number of events (%)		Multivariable hazard ratio (95% CI)	*p* value	
Baseline heart rate; hazard ratio per 10 bpm higher								
Mortality or heart failure hospitalisation	323 (31.9)	1.02 (0.96–1.08)	0.60		231 (43.2)	0.91 (0.86–0.96)	**0.001**	**0.011**
Mortality	212 (20.9)	0.97 (0.90–1.05)	0.50		112 (20.9)	0.96 (0.89–1.04)	0.40	0.75
HF hospitalisation^a^	198 (19.5)	1.02 (0.94–1.11)	0.62		139 (26.0)	0.95 (0.88–1.02)	0.13	0.20

Bold values indicate *p* < 0.05

Multivariable model adjusted for the BIOSTAT-CHF risk prediction model

BIOSTAT-CHF risk prediction model for combined endpoint of Mortality and HF hospitalisation: age, HF hospitalisation in the previous year, peripheral oedema, systolic blood pressure, log-NT-proBNP, haemoglobin, HDL cholesterol, sodium, beta-blocker use at baseline

BIOSTAT-CHF risk prediction model for heart failure hospitalisation alone: age, previous HF hospitalisation, presence of oedema, systolic blood pressure and estimated glomerular filtration rate

BIOSTAT-CHF risk prediction model for mortality alone: age, blood urea nitrogen, NT-proBNP, haemoglobin and beta-blocker use at baseline

^a^Competing risk of death

Baseline heart rate was not a significant predictor of the primary outcome in SR patients (HR per 10 bpm higher: 1.02 95% CI 0.96–1.08, *p* = 0.60), however, higher baseline heart rate was significantly associated with improved outcome in patients with AF (HR per 10 bpm higher: 0.91; 95% CI 0.86–0.96, *p* = 0.001; *p* for interaction vs. sinus rhythm 0.011). There were no significant associations for the individual endpoints of mortality and HF hospitalisation (Table 2).

### Relationship between achieved heart rate at 9 months, change in heart rate at 9 months and outcome

ECGs at the 9-month visit were available for 1155 patients. 198 patients died prior to their 9 month visit, while 195 patients did not have an ECG available. After exclusion of 125 patients with paced rhythms, 1030 patients remained for analysis, of which 734 (71.3%) were in sinus rhythm and 296 (28.7%) were in AF. Heart rate-lowering medication use at 9 months is shown in Table 3. AF at the 9-month ECG was associated with increased likelihood of the primary outcome compared to SR when added to the BIOSTAT risk prediction model (HR 1.63; 95% CI 1.18–2.23, *p* = 0.003).

**Table 3:** Heart rate controlling medication prescription at 9 months

	Sinus rhythm (734)	Atrial fibrillation (296)
Beta-blocker	691 (94.1)	276 (93.2)
Digoxin	208 (28.3)	201 (67.9)
Verapamil/diltiazem	8 (1.1)	8 (2.7)

Mean achieved heart rate at 9 months was significantly lower in SR patients compared to AF (67 ± 13 versus 81 ± 18 bpm, respectively, *p* < 0.001). Higher baseline heart was significantly associated with a greater reduction in heart rate at 9 months (*r* = − 0.77, *p* < 0.001) and an increase in beta-blocker dose at 9 months (*r* = 0.12, *p* < 0.001). After adjustment for the BIOSTAT risk prediction model and likelihood of uptitration, a higher achieved heart rate at 9 months was significantly associated with increased likelihood of the primary outcome in SR patients (HR 1.26 per 10 bpm higher; 95% CI 1.10–1.46, *p* = 0.001) but not in AF (HR 1.08 per 10 bpm higher; 95% CI 0.94–1.23, *p* = 0.18, *p* for interaction vs. SR 0.26) (Table 4). There were no significant associations between achieved heart rate and the individual endpoints.

**Table 4:** Cox regression analyses of achieved heart rate and change in heart rate at 9 months on clinical outcomes

	Sinus rhythm (*n* = 734)			Atrial fibrillation (*n* = 296)			Interaction *p* value
	Number of events (%)	Multivariable hazard ratio (95% CI)	*p* value	Number of events (%)	Multivariable hazard ratio (95% CI)	*p* value	
Achieved heart rate; hazard ratio per 10 bpm higher^a^							
Mortality or heart failure hospitalisation	168 (22.9)	1.29 (1.10–1.46)	**0.001**	115 (38.9)	1.08 (0.94–1.23)	0.18	0.26
Mortality		1.00 (0.87–1.15)	0.96		1.02 (0.88–1.18)	0.77	0.20
HF hospitalisation^+^		1.07 (0.91–1.27)	0.42		0.84 (0.65–1.07)	0.16	0.99
Change in heart rate; hazard ratio per 10 bpm decrease^a^							
Mortality or heart failure hospitalisation	168 (22.9)	0.83 (0.75–0.91)	< **0.001**	115 (38.9)	0.89 (0.81–0.98)	**0.018**	0.97
Mortality		0.95 (0.88–1.03)	0.23		0.92 (0.84–1.02)	0.11	0.50
HF hospitalisation^b^		0.88 (0.77–1.00)	**0.046**		0.93 (0.85–1.01)	0.10	0.91

Bold values indicate *p* < 0.05

BIOSTAT-CHF risk prediction model for mortality and HF hospitalisation includes: age, HF hospitalisation in the previous year, peripheral oedema, systolic blood pressure, NT-proBNP, haemoglobin, HDL cholesterol, sodium, beta-blocker use at baseline

BIOSTAT-CHF risk prediction model for heart failure hospitalisation alone: age, previous HF hospitalisation, presence of oedema, systolic blood pressure and estimated glomerular filtration rate

BIOSTAT-CHF risk prediction model for mortality alone: age, blood urea nitrogen, NT-proBNP, haemoglobin and beta-blocker use at baseline

^a^Adjusted for likelihood of uptitraton and BIOSTAT-CHF risk prediction model

^b^Competing risk of death

There was no significant difference in change in heart rate at 9 months between SR and AF patients (− 11.5 ± 21.9 bpm versus − 9.1 ± 25.9 bpm, respectively; *p* = 0.12). In multivariable analysis, a decrease in heart rate was significantly associated with reduced likelihood of the primary outcome in both SR and AF (SR: HR 0.83 per 10 bpm decrease; 95% CI 0.75–0.91, *p* < 0.001; AF: HR 0.89 per 10 bpm decrease; 95% CI 0.81–0.98, *p* = 0.018, *p* for interaction vs. SR 0.97) (Table 4). A decrease in heart rate at 9 months was also significantly associated with reduced HF hospitalisation in patients in SR (HR 0.88 per 10 bpm decrease; 95% CI 0.77–1.00, *p* = 0.046).

### Effects of changes in heart rate in patients stratified by baseline heart rate

**Fig. 2:**
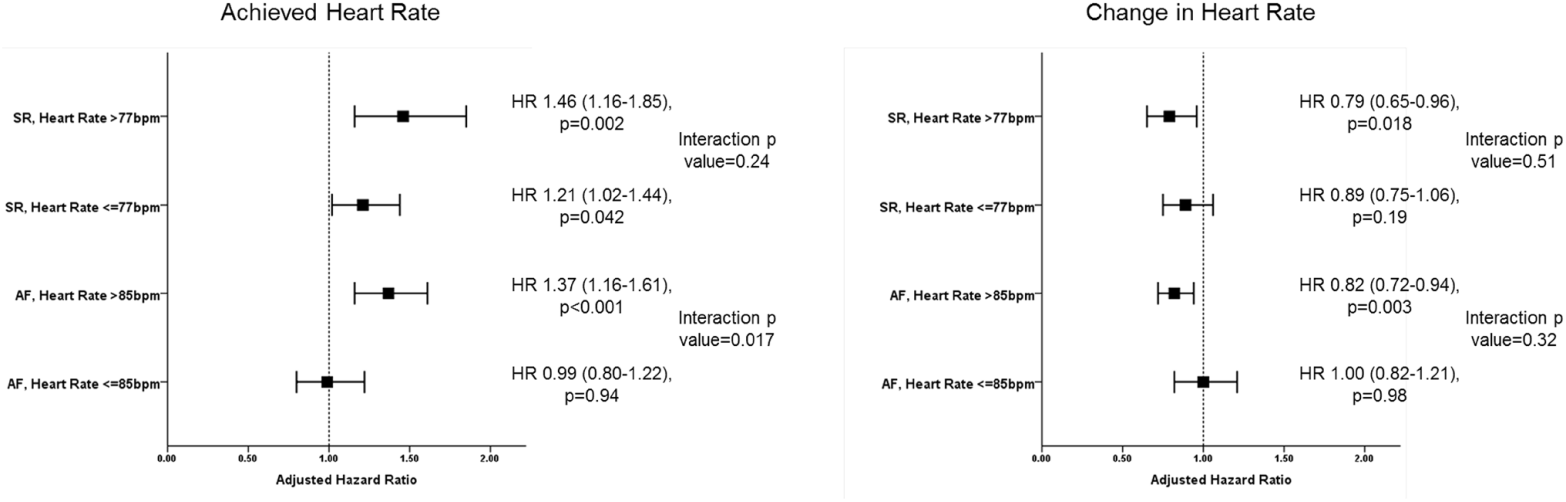
The relationship between achieved heart rate and change in heart rate at 9 months stratified by baseline heart rate. Association of achieved heart rate (left) and change in heart rate (right) with the primary outcome in sinus rhythm and atrial fibrillation stratified by baseline heart rate above and below the median

Among the patients assessed at 9 months, baseline heart rate was 77 bpm in those in SR and 85 bpm in those in AF. Higher achieved heart rate and change in heart rate were significantly associated with outcome regardless of baseline heart rate in sinus rhythm (Fig. 2), however, a different pattern was seen in patients in AF however, with higher achieved heart rate only being associated with worse outcome in patients with higher baseline heart rates (baseline heart rate > 85 bpm: HR 1.37 per 10 bpm higher; 95% CI 1.16–1.61, *p* < 0.001; ≤ 85 bpm: HR 0.99 per 10 bpm higher; 95% CI 0.80–1.22, *p* = 0.94, *p* for interaction 0.017) (Fig. 2). A similar pattern was seen with change in heart rate.

## Discussion

In this multi-national, multi-centre contemporary study of HF patients with left ventricular systolic dysfunction on sub-optimal doses of beta-blocker therapy subjected to treatment intensification, we found that both achieved heart rate and change in heart rate at 9 months are strongly associated with outcome in HFrEF patients in SR regardless of baseline resting heart rate. In contrast, only a decrease in heart rate was significantly associated with improved outcome in AF patients, and in particular only in those with higher baseline heart rates.

HF and AF frequently co-exist and present an additional layer of complexity in management [24]. Established markers of prognosis, such as baseline heart rate, and established therapies such as beta-blockers, appear to be less effective in HF patients in AF compared to those in SR. Our results align with the increasing evidence from observational studies [3, 4], randomised controlled trials [7] and meta-analysis [5] that suggests that baseline heart rate is not an important prognostic marker in HFrEF patients in AF. Very few studies however have examined the prognostic significance of follow-up heart rate in patients with HFrEF in SR and in AF, particularly in the setting of treatment change. Cullington et al. found that heart rate at 1 year was a significant independent predictor of outcome in SR patients but not in AF [3], while in contrast, in an analysis of the Candesartan in heart failure: assessment of reduction in mortality and morbidity (CHARM) programme, Vazir et al. found that change in heart rate was also an independent predictor of poor outcome in both SR and AF patients, although the prognostic significance was less in AF patients [7]. Our study differs from these, however, as we have evaluated a cohort of patients who were not receiving target doses of beta-blockers. A recent large meta-analysis of beta-blocker HF trials reported that a lower achieved heart rate was associated with improved outcome only in SR patients [25]. It is noteworthy that many of these beta-blocker trials were conducted in patients that had not been treated with contemporary heart failure therapy. Our study provides new evidence involving contemporary clinical practice.

A major part of our study was to examine the effect of a change in heart rate in conjunction with changes in beta-blocker dose. We found that despite similar reductions in heart rate and similar increase in beta-blocker dose, the prognostic significance of both achieved and changes in heart rate were only seen in AF patients at higher baseline heart rates. It is not completely clear why heart rate reduction using beta-blockers should not be associated with improved outcome in HFrEF patients in AF regardless of baseline heart rate, as it is in SR. It has been postulated that higher heart rates in patients with AF is beneficial to compensate for the loss of atrial ejection and reduced left ventricular diastolic filling [26]. It may also be that reduction in heart rate using increased dosages of beta-blockade is not a beneficial strategy, perhaps due to the potential for ventricular pauses that might be associated with adverse outcome [27].

We found however that there were benefits in targeting heart rate in AF patients with higher baseline heart rate. A common clinical question in treatment of HFrEF in patients AF is whether the AF is secondary to HF or vice versa [28]. It may be that in some HFrEF patients in AF, the presence of AF may be a reflection of HF severity [29]. However, it is also possible that the AF is driving the HF, and that control of the heart rate in this setting (“tachycardiomyopathy”) might improve HF outcome [30]. This might also explain our somewhat surprising finding that increased baseline heart rate was significantly associated with improved outcome following treatment intensification over 9 months. Being aware of the fact that the median heart rate was significantly higher in patients with AF at baseline, we noted that a higher baseline heart rate was significantly associated with an increase in beta-blocker dose (i.e., more likelihood of uptitration) and a greater reduction in heart rate at 9 months. While we cannot determine causality due to the nature of our study, this does suggest that potentially some of these patients at higher baseline heart rate may have benefited from intensified therapy and may reflect an element of “tachycardiomyopathy”. While it is often difficult to diagnose tachycardiomyopathy prospectively, this might account for this unexpected finding.

Heart rate reduction by other mechanisms generally appears to have limited benefit in AF-HFrEF patients. Digoxin is, at best, neutral in terms of clinical outcome in AF patients with HFrEF, though it might provide some symptomatic benefit, while non-dihyrdopyridine calcium channel blockers are contra-indicated in HF [31]. Alternative strategies may be more beneficial. There may be a role for AV nodal ablation and cardiac resynchronisation device implantation, however, no large randomised trials have been conducted to confirm this as yet [32]. Another strategy that has been proposed is AF ablation with recent data reporting improved outcomes in patients with AF and HFrEF [33]. Indeed, these results are particularly prescient given the recent results of the CASTLE-AF trial [33], as they suggest that persisting with beta-blocker dose uptitration to maximal targets with the aim of lowering heart rate may not provide any mortality benefit in HFrEF patients in AF, and perhaps other strategies such as pulmonary vein isolation or pacemaker implantation and AV node ablation may prove to have more prognostic benefit to remove the burden of AF.

Our study has some limitations. First, this is a post hoc analysis of a prospective study. However, one of the strengths of the study was that as well as being an observational study, the protocol also mandated uptitration of HF therapy, thus adding some of the benefits of a clinical trial element. Second, we only obtained resting heart rhythm and rate at two separate time points. It is possible that patients may have been in paroxysmal AF at the time of their visit, while in SR the majority of time in the interim or vice versa. Further insights into the effect of heart rate on prognosis may have been obtained by more frequent heart rate monitoring. Third, we did not have any information on changes in heart rate or beta-blocker dose beyond 9 months, which might have had an impact on clinical outcomes. Additionally, despite the overall size of this study, there were a relatively low number of patients in AF at 9 months, thus we cannot exclude that interactions may have become significant with larger numbers. Further studies specifically examining beta-blocker uptitration are required to confirm these findings. Finally, due to the number of patients, we did not further stratify the cohort based on cardio-selectivity of prescribed beta-blocker. Larger cohorts should be evaluated with the specific aim of determining whether heart rate reduction mediated by cardio-selective beta-blockers is more beneficial in AF patients with HFrEF.

## Conclusions

In HFrEF patients in SR both achieved and change in heart rate following beta-blocker uptitration were associated with improved outcomes, regardless of heart rate at baseline.

Despite a similar increase in beta-blocker dose and baseline heart rate reduction in HFrEF patients in AF, achieved and decrease in heart rate from baseline were only prognostically significant in patients with higher baseline heart rates.

## References

[1] Swedberg K, Komajda M, Bohm M, Borer JS, Ford I, Dubost-Brama A, Lerebours G, Tavazzi L (2010). Ivabradine and outcomes in chronic heart failure (SHIFT): a randomised placebo-controlled study. Lancet.

[2] Triposkiadis F, Karayannis G, Giamouzis G, Skoularigis J, Louridas G, Butler J (2009). The sympathetic nervous system in heart failure physiology, pathophysiology, and clinical implications. J Am Coll Cardiol.

[3] Cullington D, Goode KM, Zhang J, Cleland JG, Clark AL (2014). Is heart rate important for patients with heart failure in atrial fibrillation?. JACC Heart Fail.

[4] Li SJ, Sartipy U, Lund LH, Dahlstrom U, Adiels M, Petzold M, Fu M (2015). Prognostic significance of resting heart rate and use of beta-blockers in atrial fibrillation and sinus rhythm in patients with heart failure and reduced ejection fraction: findings from the Swedish Heart Failure Registry. Circ Heart Fail.

[5] Simpson J, Castagno D, Doughty RN, Poppe KK, Earle N, Squire I, Richards M, Andersson B, Ezekowitz JA, Komajda M, Petrie MC, McAlister FA, Gamble GD, Whalley GA, McMurray JJ (2015). Is heart rate a risk marker in patients with chronic heart failure and concomitant atrial fibrillation? Results from the MAGGIC meta-analysis. Eur J Heart Fail.

[6] Van Gelder IC, Groenveld HF, Crijns HJ, Tuininga YS, Tijssen JG, Alings AM, Hillege HL, Bergsma-Kadijk JA, Cornel JH, Kamp O, Tukkie R, Bosker HA, Van Veldhuisen DJ, Van den Berg MP (2010). Lenient versus strict rate control in patients with atrial fibrillation. N Engl J Med.

[7] Vazir A, Claggett B, Jhund P, Castagno D, Skali H, Yusuf S, Swedberg K, Granger CB, McMurray JJ, Pfeffer MA, Solomon SD (2015). Prognostic importance of temporal changes in resting heart rate in heart failure patients: an analysis of the CHARM program. Eur Heart J.

[8] Hamill V, Ford I, Fox K, Bohm M, Borer JS, Ferrari R, Komajda M, Steg PG, Tavazzi L, Tendera M, Swedberg K (2015). Repeated heart rate measurement and cardiovascular outcomes in left ventricular systolic dysfunction. Am J Med.

[9] Ponikowski P, Voors AA, Anker SD, Bueno H, Cleland JG, Coats AJ, Falk V, Gonzalez-Juanatey JR, Harjola VP, Jankowska EA, Jessup M, Linde C, Nihoyannopoulos P, Parissis JT, Pieske B, Riley JP, Rosano GM, Ruilope LM, Ruschitzka F, Rutten FH, van der Meer P (2016). 2016 ESC Guidelines for the diagnosis and treatment of acute and chronic heart failure: the Task Force for the diagnosis and treatment of acute and chronic heart failure of the European Society of Cardiology (ESC). Developed with the special contribution of the Heart Failure Association (HFA) of the ESC. Eur J Heart Fail.

[10] Frohlich H, Torres L, Tager T, Schellberg D, Corletto A, Kazmi S, Goode K, Grundtvig M, Hole T, Katus HA, Cleland JGF, Atar D, Clark AL, Agewall S, Frankenstein L (2017). Bisoprolol compared with carvedilol and metoprolol succinate in the treatment of patients with chronic heart failure. Clin Res Cardiol.

[11] Porapakkham P, Porapakkham P, Krum H (2010). Is target dose of beta-blocker more important than achieved heart rate or heart rate change in patients with systolic chronic heart failure?. Cardiovasc Ther.

[12] McAlister FA, Wiebe N, Ezekowitz JA, Leung AA, Armstrong PW (2009). Meta-analysis: beta-blocker dose, heart rate reduction, and death in patients with heart failure. Ann Intern Med.

[13] Cullington D, Goode KM, Clark AL, Cleland JG (2012). Heart rate achieved or beta-blocker dose in patients with chronic heart failure: which is the better target?. Eur J Heart Fail.

[14] Metra M, Torp-Pedersen C, Swedberg K, Cleland JG, Di Lenarda A, Komajda M, Remme WJ, Lutiger B, Scherhag A, Lukas MA, Charlesworth A, Poole-Wilson PA (2005). Influence of heart rate, blood pressure, and beta-blocker dose on outcome and the differences in outcome between carvedilol and metoprolol tartrate in patients with chronic heart failure: results from the COMET trial. Eur Heart J.

[15] Fiuzat M, Wojdyla D, Pina I, Adams K, Whellan D, O’Connor CM (2016). Heart rate or beta-blocker dose? Association with outcomes in ambulatory heart failure patients with systolic dysfunction: results from the HF-ACTION trial. JACC Heart Fail.

[16] Swedberg K, Komajda M, Bohm M, Borer J, Robertson M, Tavazzi L, Ford I (2012). Effects on outcomes of heart rate reduction by ivabradine in patients with congestive heart failure: is there an influence of beta-blocker dose?: findings from the SHIFT (Systolic Heart failure treatment with the I(f) inhibitor ivabradine Trial) study. J Am Coll Cardiol.

[17] Fu M, Ahrenmark U, Berglund S, Lindholm CJ, Lehto A, Broberg AM, Tasevska-Dinevska G, Wikstrom G, Agard A, Andersson B (2017). Adherence to optimal heart rate control in heart failure with reduced ejection fraction: insight from a survey of heart rate in heart failure in Sweden (HR-HF study). Clin Res Cardiol.

[18] Kotecha D, Holmes J, Krum H, Altman DG, Manzano L, Cleland JG, Lip GY, Coats AJ, Andersson B, Kirchhof P, von Lueder TG, Wedel H, Rosano G, Shibata MC, Rigby A, Flather MD, Beta-Blockers in Heart Failure Collaborative G (2014). Efficacy of beta blockers in patients with heart failure plus atrial fibrillation: an individual-patient data meta-analysis. Lancet.

[19] Rienstra M, Damman K, Mulder BA, Van Gelder IC, McMurray JJ, Van Veldhuisen DJ (2013). Beta-blockers and outcome in heart failure and atrial fibrillation: a meta-analysis. JACC Heart Fail.

[20] Corletto A, Frohlich H, Tager T, Hochadel M, Zahn R, Kilkowski C, Winkler R, Senges J, Katus HA, Frankenstein L (2018). Beta blockers and chronic heart failure patients: prognostic impact of a dose targeted beta blocker therapy vs. heart rate targeted strategy. Clin Res Cardiol.

[21] Voors AA, Anker SD, Cleland JG, Dickstein K, Filippatos G, van der Harst P, Hillege HL, Lang CC, Ter Maaten JM, Ng L, Ponikowski P, Samani NJ, van Veldhuisen DJ, Zannad F, Zwinderman AH, Metra M (2016). A systems BIOlogy Study to TAilored Treatment in Chronic Heart Failure: rationale, design, and baseline characteristics of BIOSTAT-CHF. Eur J Heart Fail.

[22] Ponikowski P, Voors AA, Anker SD, Bueno H, Cleland JG, Coats AJ, Falk V, Gonzalez-Juanatey JR, Harjola VP, Jankowska EA, Jessup M, Linde C, Nihoyannopoulos P, Parissis JT, Pieske B, Riley JP, Rosano GM, Ruilope LM, Ruschitzka F, Rutten FH, van der Meer P (2016). 2016 ESC Guidelines for the diagnosis and treatment of acute and chronic heart failure: the Task Force for the diagnosis and treatment of acute and chronic heart failure of the European Society of Cardiology (ESC)Developed with the special contribution of the Heart Failure Association (HFA) of the ESC. Eur Heart J.

[23] Ouwerkerk W, Voors AA, Anker SD, Cleland JG, Dickstein K, Filippatos G, van der Harst P, Hillege HL, Lang CC, Ter Maaten JM, Ng LL, Ponikowski P, Samani NJ, van Veldhuisen DJ, Zannad F, Metra M, Zwinderman AH (2017). Determinants and clinical outcome of uptitration of ACE-inhibitors and beta-blockers in patients with heart failure: a prospective European study. Eur Heart J.

[24] Bristow MR, Aleong RG (2013). Treatment of the heart failure patient with atrial fibrillation: a major unmet need. JACC Heart Fail.

[25] Kotecha D, Flather MD, Altman DG, Holmes J, Rosano G, Wikstrand J, Packer M, Coats AJS, Manzano L, Bohm M, van Veldhuisen DJ, Andersson B, Wedel H, von Lueder TG, Rigby AS, Hjalmarson A, Kjekshus J, Cleland JGF, Beta-Blockers in Heart Failure Collaborative (2017). Heart rate, heart rhythm, and prognostic benefits of beta-blockers in heart failure: individual patient-data meta-analysis. J Am Coll Cardiol.

[26] Deedwania PC, Lardizabal JA (2010). Atrial fibrillation in heart failure: a comprehensive review. Am J Med.

[27] Mareev Y, Cleland JG (2015). Should beta-blockers be used in patients with heart failure and atrial fibrillation?. Clin Ther.

[28] Cadrin-Tourigny J, Shohoudi A, Roy D, Talajic M, Tadros R, Mondesert B, Dyrda K, Rivard L, Andrade JG, Macle L, Guerra PG, Thibault B, Dubuc M, Khairy P (2017). decreased mortality with beta-blockers in patients with heart failure and coexisting atrial fibrillation: an AF-CHF substudy. JACC Heart Fail.

[29] Smit MD, Moes ML, Maass AH, Achekar ID, Van Geel PP, Hillege HL, van Veldhuisen DJ, Van Gelder IC (2012). The importance of whether atrial fibrillation or heart failure develops first. Eur J Heart Fail.

[30] Nerheim P, Birger-Botkin S, Piracha L, Olshansky B (2004). Heart failure and sudden death in patients with tachycardia-induced cardiomyopathy and recurrent tachycardia. Circulation.

[31] van Veldhuisen DJ, Van Gelder IC, Ahmed A, Gheorghiade M (2013). Digoxin for patients with atrial fibrillation and heart failure: paradise lost or not?. Eur Heart J.

[32] Gasparini M, Leclercq C, Lunati M, Landolina M, Auricchio A, Santini M, Boriani G, Lamp B, Proclemer A, Curnis A, Klersy C, Leyva F (2013). Cardiac resynchronization therapy in patients with atrial fibrillation: the CERTIFY study (Cardiac Resynchronization Therapy in Atrial Fibrillation Patients Multinational Registry). JACC Heart Fail.

[33] Marrouche NF, Brachmann J, Andresen D, Siebels J, Boersma L, Jordaens L, Merkely B, Pokushalov E, Sanders P, Proff J, Schunkert H, Christ H, Vogt J, Bansch D (2018). Catheter ablation for atrial fibrillation with heart failure. N Engl J Med.

